# Supplementary material for: Empirical methods for controlling false positives and estimating confidence in ChIP-Seq peaks
Source: BMC Bioinformatics. 2008 Dec 5;9:523. doi: 10.1186/1471-2105-9-523 (PMC2628906; doi:10.1186/1471-2105-9-523)
Supplement: Additional File 1 — A variety of html documents from the USeq web site detailing the available applications, their best usage, output file type descriptions, command line menus, etc. [file 1471-2105-9-523-S1.zip › USeqUserGuides/JavaDoc.zip!/JavaDoc/gata/main/PlotterPreferences.html]

PlotterPreferences


|  |  |  |
| --- | --- | --- |
| **Overview** \| **Package** \| **Class** \| **Use** \| **Tree** \| **Deprecated** \| **Index** \| **Help** | |  |
| **PREV CLASS**   **NEXT CLASS** | **FRAMES**    **NO FRAMES**     **All Classes** |  |
| SUMMARY: NESTED \| FIELD \| CONSTR \| METHOD | DETAIL: FIELD \| CONSTR \| METHOD |  |


---


## gata.main Class PlotterPreferences

```
java.lang.Object
  gata.main.PlotterPreferences
```

**All Implemented Interfaces:**: java.io.Serializable

---

``` public class PlotterPreferences extends java.lang.Object implements java.io.Serializable ```

**Author:**
:   nix
    Used to set GATAPlotter preferences upon booting, saved upon close.

**See Also:**: Serialized Form

---

**Constructor Summary**

`PlotterPreferences()`


| **Method Summary** | |
| --- | --- |
| `void` | `fetchAndAssignGATAParams(GATAParams p)` |
| `void` | `setAnnoScaleDefaults(AnnoSpecParams asp)` |
| `void` | `setCurrentPlotterPreferences(GATAParams p)`             Contains all the default preferences for GATAParams |
| `void` | `setPlotterDefaults(GATAParams p)`             Contains all the default preferences for GATAParams, alter these to change the default view |

**Methods inherited from class java.lang.Object**

`equals, getClass, hashCode, notify, notifyAll, toString, wait, wait, wait`

**Constructor Detail**

### PlotterPreferences

```
public PlotterPreferences()
```


**Method Detail**

### fetchAndAssignGATAParams

```
public void fetchAndAssignGATAParams(GATAParams p)
```

---


### setAnnoScaleDefaults

```
public void setAnnoScaleDefaults(AnnoSpecParams asp)
```

---


### setPlotterDefaults

```
public void setPlotterDefaults(GATAParams p)
```

:   Contains all the default preferences for GATAParams, alter these to change the default view

---


### setCurrentPlotterPreferences

```
public void setCurrentPlotterPreferences(GATAParams p)
```

:   Contains all the default preferences for GATAParams


---


|  |  |  |
| --- | --- | --- |
| **Overview** \| **Package** \| **Class** \| **Use** \| **Tree** \| **Deprecated** \| **Index** \| **Help** | |  |
| **PREV CLASS**   **NEXT CLASS** | **FRAMES**    **NO FRAMES**     **All Classes** |  |
| SUMMARY: NESTED \| FIELD \| CONSTR \| METHOD | DETAIL: FIELD \| CONSTR \| METHOD |  |


---
